# Tailoring a training based on the *Mental Health Gap Action Programme (mhGAP) Intervention Guide (IG)* to Tunisia: process and relevant adaptations

**DOI:** 10.1017/gmh.2018.8

**Published:** 2018-05-09

**Authors:** Jessica Spagnolo, François Champagne, Nicole Leduc, Wahid Melki, Imen Guesmi, Nesrine Bram, Ann-Lise Guisset, Myra Piat, Marc Laporta, Fatma Charfi

**Affiliations:** 1 School of Public Health; Institut de recherche en santé publique de l'Université de Montréal (IRSPUM), University of Montreal, 7101 Parc Avenue, Montreal, Québec, H3N 1X9, Canada; 2 Montreal WHO-PAHO Collaborating Center for Research and Training in Mental Health, 6875 LaSalle boul., Montreal, Québec, H4H 1R3, Canada; 3 Razi Hospital, Cité des Orangers, Manouba, Tunis, Tunisia; 4 Faculty of Medicine, University of Tunis El-Manar, 94 Rommana 1068, Tunis, Tunisia; 5 Centre médico et universitaire de Manouba, Tunisia; 6 World Health Organization Headquarters, Avenue Appia 20, CH-1211 Geneva 27; 7 Douglas Mental Health University Institute (The Montreal West Island Integrated University Health and Social Services Center), 6875 LaSalle boul., Montreal, Québec, H4H 1R3, Canada; 8 McGill University, 845 Sherbrooke Street West Montreal, Québec, H3A 0G4, Canada; 9 Mongi-Slim Hospital, 2046 Sidi Daoud, La Marsa, Tunisia

**Keywords:** Adaptation, mhGAP training, mental health, teaching and learning, Tunisia

## Abstract

**Background:**

In order to make mental health services more accessible, the Tunisian Ministry of Health, in collaboration with the School of Public Health at the University of Montreal, the World Health Organization office in Tunisia and the Montreal World Health Organization-Pan American Health Organization Collaborating Center for Research and Training in Mental Health, implemented a training programme based on the *Mental Health Gap Action Programme (mhGAP) Intervention Guide (IG) (version 1.0)*, developed by the World Health Organization. This article describes the phase prior to the implementation of the training, which was offered to general practitioners working in primary care settings in the Greater Tunis area of Tunisia.

**Methods:**

The phase prior to implementation consisted of adapting the standard *mhGAP-IG (version 1.0)* to the local primary healthcare context. This adaptation process, an essential step before piloting the training, involved discussions with stakeholder groups, as well as field observations.

**Results:**

Through the adaptation process, we were able to make changes to the standard training format and material. In addition, the process helped uncover systemic barriers to effective mental health care.

**Conclusions:**

Targeting these barriers in addition to implementing a training programme may help reduce the mental health treatment gap, and promote implementation that is successful and sustainable.

## Introduction

Tunisia faces many challenges related to mental health care. First, it is estimated that roughly 1% of the country's total public-sector healthcare budget is allocated to mental health, an area affecting a substantial portion of the population (World Health Organization and Ministry of Health Tunisia, 2008; Unité de Promotion de la Santé Mentale, 2013). It is important to note, however, that this amount is lower than the estimated average of 1.9% allocated to mental health in other lower and middle-income countries (World Health Organization, 2013a; Mackenzie & Kesner, 2016). Moreover, of this 1% budget, half is used to sustain hospital settings treating mental illness, to the detriment of developing community-based mental health services (World Health Organization and Ministry of Health Tunisia, 2008). Second, there is a deficit of mental healthcare professionals (Bruckner *et al.* 2011) and they are inequitably distributed across the country. For example, mental healthcare professionals mainly work in and around the capital, or along the coastline (World Health Organization and Ministry of Health Tunisia, 2008; Unité de Promotion de la Santé Mentale, 2013), despite much-needed services within the interior of the country (Unité de Promotion de la Santé Mentale, 2013). Third, there are challenges related to the integration of mental health at the primary care level namely due to deficits in mental health training and remaining echoes of stigma against mental illness (World Health Organization, 2013a). Lack of integration and effects of stigmatization encourage the use of the only standing and already overly crowded mental health hospital, as well as the limited psychiatric units located within general hospitals (Unité de Promotion de la Santé Mentale, 2013). With the burden caused by mental disorders, substance use disorders and suicide anticipated to increase given economic unrest in the country (Unité de Promotion de la Santé Mentale, 2013; Charara *et al.* 2017), barriers to effective and accessible mental health care are generating concern.

To address these challenges in the country, general practitioners (GPs) working at the primary care level are targets of health system reform given their strategic position in the healthcare system (Unité de Promotion de la Santé Mentale, 2013; Spagnolo *et al.* 2017). However, despite an estimated one-third of their consultations being related to mental health (Melki *et al.* 2003; Unité de Promotion de la Santé Mentale, 2013; World Health Organization, 2016), GPs continue to lack specific knowledge and skills to adequately address mental health-related issues (Unité de Promotion de la Santé Mentale, 2013). For example, it has been reported that most GPs have insufficient mental health training, a lack of mastery over the prescription of psychotropic medications, and a fear of treating mental illness (Melki *et al.* 2003; Unité de Promotion de la Santé Mentale, 2013). For these reasons, the Tunisian Ministry of Health (more specifically, the Presidents of the Committee for Mental Health Promotion and Technical Committee Against Suicide), in collaboration with the School of Public Health at the University of Montreal, the World Health Organization office in Tunisia and the Montreal World Health Organization-Pan American Health Organization Collaborating Center for Research and Training in Mental Health, implemented a training based on the *Mental Health Gap Action Programme (mhGAP) Intervention Guide (IG) (version 1.0*) (World Health Organization, 2010), developed by the World Health Organization. The goal of the training is to assist in the delivery of effective mental health services by non-specialists, for conditions considered of high priority in low- and middle-income countries (World Health Organization, 2010; Eaton *et al.* 2011).

It is important to note that the *mhGAP-IG* and accompanying training content are standardized tools developed for use in a wide range of low- and middle-income countries to address the alarming mental health treatment gap (World Health Organization, 2010; Lund *et al.* 2012). Within these countries, however, lie differences in the conception of mental health conditions and mental healthcare organization, factors that encourage varying socio-cultural contexts (Abdulmalik *et al.* 2013; Thornicroft & Patel, 2014). Therefore, given the heterogeneity of low- and middle-income countries, the standard *mhGAP-IG*, training material and programme require adaptation before implementation (World Health Organization, 2010).

Since 2010, trainings based on the *mhGAP-IG* have been implemented in over 90 countries (Keynejad *et al*. 2017). Evidence has emerged over the past years of the programme's effectiveness at improving non-specialists’ detection, treatment and management of mental health conditions within primary and community-based settings (Keynejad *et al*. 2017). However, what is currently scarcer is knowledge on ‘how’ this complex intervention is adapted to specific settings as to make it culturally appropriate, and therefore useful. Generating such evidence is a current priority in global mental health, as it can aid in the sustainability and scale-up of the programme (Eaton *et al.* 2011; Thornicroft & Patel, 2014), as well as empower local stakeholders to take ownership of the implementation process.

The purpose of this article is to describe the phase prior to the implementation of a mental health training programme based on the *mhGAP-IG (version 1.0)* in the Greater Tunis area of Tunisia, and report on adaptations made to the standard training material and programme, essential before piloting. This project is part of a larger trial, seeking to evaluate the training programme implemented in the Greater Tunis area using a randomized controlled trial and implementation analysis. Competencies evaluated pre- and post-training include mental health knowledge, attitudes towards mental illness and the field of mental health, self-efficacy in detecting, treating and managing mental illness in primary care, as well as clinical practice in mental health (Spagnolo *et al.* 2017).

## Methods

Preparing for the implementation of a mental health training programme based on the *mhGAP-IG (version 1.0)* began in September 2015, and necessitated multiple steps. The first step consisted of identifying mental health needs or gaps in the Greater Tunis area by: (1) using the *Adaptation Guide*, a tool developed by the World Health Organization to accompany the *mhGAP-IG*; (2) discussing with members of the Ministry of Health; and (3) consulting epidemiological studies on mental health trends, post-Tunisian Revolution of 2010–2011. The second step in preparing for implementation consisted of developing a preliminary training programme and schedule, tailored to the Greater Tunis area. The last step prior to implementation consisted of conducting field observations in primary healthcare clinics.

### Step 1: identifying mental health needs

Three Tunisian psychiatrists were appointed by members of the Tunisian Ministry of Health as trainers given their expertise in mental health organization, and familiarity with the functioning of both institutional- and community-based mental health services in the Greater Tunis area. Using the *Adaptation Guide* as a roadmap for dialogue, three group discussions were conducted with the trainer-psychiatrists on language used in training material, context's impact on training content (including conditions’ specificities and the use of psychotherapy), availability of medication at the level of primary care, and availability of community-based mental health services. These discussions were important not only to aid in the adaptation of standard training material, but also to understand the types of resources (i.e. pharmacological, human and/or organizational) missing in the Greater Tunis area, as compared with the suggested, standard resources listed in the *mhGAP-IG (version 1.0).*

Discussions with members of the Ministry of Health validated the findings uncovered using the *Adaptation Guide*, and allowed us to further understand the current trends in mental disorders, substance use disorders and suicide. These trends were also confirmed by consulting the limited epidemiological studies on mental disorders, substance use disorders and suicide, especially post-Revolution in Tunisia. In addition, discussions with members of the Ministry of Health highlighted GPs’ available referral network for mental disorders, substance use disorders and suicide, and how it may be adapted for the purposes of the training.

### Step 2: developing a preliminary training programme and schedule

The next step in preparing for the implementation of the *mhGAP-IG (version 1.0)* consisted of developing a preliminary training programme and schedule, tailored to the Greater Tunis area. This preliminary programme and schedule was developed as a collaborative effort between members of the Ministry of Health in Tunisia (WM, FC), the School of Public Health at the University of Montreal (JS, FC, NL), the Montreal World Health Organization-Pan American Health Organization Collaborating Center for Research and Training in Mental Health (ML) and the World Health Organization office in Tunisia (ALG). This training programme and schedule was presented to the three trainer-psychiatrists and seven GPs in charge of continuing medical education in the Greater Tunis area, for comments and suggestions. Members of the Tunisian Ministry of Health enlisted GPs responsible for continuing medical education because they are well-versed in mental health knowledge and skills, and would be able to assist trainer-psychiatrists during and post-training. Both trainer-psychiatrists and the seven GPs in charge of continuing medical education in the Greater Tunis area participated in a *Training of Trainers*, as an orientation to the proper use of the *mhGAP-IG (version 1.0).*

### Step 3: conducting field observations

The last step in preparing for the implementation of the *mhGAP-IG (version 1.0)* consisted of conducting field observations, between November and December 2015. Field observations included visits to primary healthcare clinics in the Greater Tunis area.

## Results

### Required adaptation 1: selecting training modules

Rates of anxiety, depressive and substance use disorders, as well as suicide, are on the rise in Tunisia (Unité de Promotion de la Santé Mentale, 2013; Ouanes *et al.* 2014; Ben Khelil *et al.* 2016a,b; World Health Organization, 2016; Ben Khelil *et al.* 2017; Charara *et al.* 2017). First, data suggests that consultations specifically for anxiety and depression have increased post-Tunisian Revolution (Unité de Promotion de la Santé Mentale, 2013; Ouanes *et al.* 2014). Second, records show that the number of suicide deaths rose 1.8 times and self-immolation, three times during the 4 years following the Revolution (Ben Khelil *et al.* 2016a; Ben Khelil *et al.* 2017). Third, there is a recorded increase in the rates of substance use (MedSPAD Committee, 2015) and substance use disorders, specifically of opioids, cannabis, ecstasy and alcohol, and especially among people under the age of 35 (Unité de Promotion de la Santé Mentale, 2013; MedSPAD Committee, 2015). The rise in anxiety, depressive and substance use disorders, as well as suicide is argued to be associated with triggering events during the Revolution (Ouanes *et al.* 2014; Ben Khelil *et al.* 2017) and current instabilities such as difficult working and living conditions (Unité de Promotion de la Santé Mentale, 2013; Ouanes *et al.* 2014).

While records do not show a significant increase in the rise of schizophrenia since the Revolution, there is worry about potential complications associated with this disorder, even if underdiagnosed. More specifically, in Tunisia, schizophrenia has been linked with suicide and suicide attempts (Ghachem *et al.* 2009). In addition, it is reported that annual mortality rates associated with schizophrenia have increased (Ghachem *et al.* 2009).

Given this contextual knowledge, members of the Ministry of Health selected specific modules from the *mhGAP-IG (version 1.0)* to address pressing and growing needs in the country. The selected modules include depression, psychosis, self-harm/suicide and alcohol/ drug use disorders. In addition, a general introduction to the *mhGAP-IG* and the module ‘*General Principles of Care*’ were included in the training. These modules provide an overview of the programme's goal, how to use the guide in consultation and appropriate clinical practices in the field of mental health.

It is important to note that the inclusion of the ‘*General Principles of Care*’ module was reinforced by field observations. More specifically, during visits to primary healthcare clinics, JS observed that some GPs shared offices to provide care, were interrupted during consultations by waiting patients and/or answered phone calls during consultations. Thus, discussions on confidentiality and clinical practices for effective communication and interactions of healthcare professionals with people seeking mental health care needed to be discussed. In addition, trainer-psychiatrists thought it appropriate to share with trainees some effective ways to engage in active listening, and ways to respectfully and effectively probe for information about mental health problems.

While rates of anxiety disorders have increased post-Revolution and remain concerning, at the time of adaptation, the accompanying training material (i.e. PowerPoints) for the module on conditions specifically related to stress (World Health Organization, 2013b) was not available in the country's working languages: French and Tunisian Arabic. This unavailability was a major implementation barrier to a much-needed module in the country. However, anxiety disorders were covered indirectly by the depression module of the standard *mhGAP-IG* (*version 1.0*) (World Health Organization, 2010).

### Required adaptation 2: developing a training format

The training based on the *mhGAP-IG* was designed to accommodate the work schedule of participants. Given that GPs conduct clinical work between 8 h and 14 h, Monday through Saturday, and continuing medical education occurs outside of these hours, the implementation of one afternoon training session per week was thus envisioned.

Training sessions would be conducted in French, the language in which medical school is taught, and all medical staff is well-versed. The sessions, as suggested by standard material, would consist of a general lecture, learning videos and group discussions. Due to high demand for training, GPs were randomly assigned to one of three work groups prior to the implementation of the training as to facilitate role plays and discussion following the general lecture. Each group would be animated by a trainer-psychiatrist and GPs responsible for continuing medical education in the Greater Tunis area. Groups would remain the same for the entirety of the training, allowing GPs from different governorates to become acquainted and share experiences with regards to mental health care. It is important to note that it was thought best by members of the Ministry of Health, trainer-psychiatrists and GPs in charge of continuing medical education in the Greater Tunis area to translate instructions for standard role plays into Tunisian Arabic, and implement them in that language as to mirror ‘real-world’ consultation in primary healthcare clinics. Translation was facilitated by the three Tunisian trainer-psychiatrists, and trainees engaged in simulation of consultations in Tunisian Arabic.

While the World Health Organization encourages ongoing supervision after the implementation of a training based on the *mhGAP-IG*, this task would not be feasible in the Greater Tunis area given the heavy time constraints of specialists. However, a 2-h support session, in respective work groups, was envisioned 1-week post-training to encourage GPs to discuss mental health cases, under the supervision of specialists. In addition, role plays were selected from the standard introduction module by trainer-psychiatrists to help further integrate knowledge, and to answer any remaining questions on the general content of the training. These role plays were also translated into Tunisian Arabic by trainer-psychiatrists and conducted by trainees in that language, as well.

As ongoing supervision by trainer-psychiatrists would not be feasible, the goal of members of the Ministry of Health was therefore to create a realistic support network for trainees, during and post-training. This support network was created by appointing GPs in charge of continuing medical education in the Greater Tunis area as ‘tutors’. This initiative seemed appropriate for several reasons. First, seeing as tutors are already well-versed in mental health care and had participated in the *Training of Trainers* along with the trainer-psychiatrists, they would be equipped to answer participants’ mental health questions between and post-training sessions. Second, being a peer to GP trainees, tutors thoroughly understand the clinical reality in primary care and can address questions or concerns using non-specialized language. Third, given that the module on conditions specifically related to stress (World Health Organization, 2013b) could not be implemented, tutors would be able to play an instrumental role in filling this knowledge gap. Lastly, given that tutors are already involved in continuing medical education, it was feasible for them to attempt to organize, every month following training and in collaboration with their directors, mental health support sessions regrouping trainees from each governorate. These scheduled sessions would thus provide trainees with the opportunity to present and gain insight on challenging clinical cases. It is important to note that trainer-psychiatrists agreed that tutors could contact them directly should more in-depth consultation or a referral be necessary.

Discussions with members of the Ministry of Health during the adaptation process highlighted available referral networks for mental disorders, substance use disorders and suicide, as well as challenges with these networks. This information is important given that the standard *mhGAP-IG* often specifies to ‘*Consult a specialist*’. In these cases, specialists are psychiatrists, and they may be consulted primarily by referral. Referrals to specialized care are done by letter. To facilitate and accelerate referrals (if needed during the implementation of the training programme), trainer-psychiatrists provided trainees with their telephone numbers.

The training content and format, as conducted in the Greater Tunis area, are presented in Table 1.

**Table 1.** Outline of the Mental Health Gap Action Programme (mhGAP) Intervention Guide (IG) training as tailored for the Greater Tunis area (Tunisia)

Schedule	Module	Learning objectives	Training components
Week 1 (13 h30–17 h)	*Introduction* and *‘General Principles of Care’*	*To learn*: about the mental health treatment gap in low- and middle-income countries (and thus the need to develop the *mhGAP*); how to use the *mhGAP-IG*; about effective clinical practices in mental health	*13 h30–16 h*: Welcome and general lecture using *mhGAP-IG* PowerPoint: introduction to the programme; general principles of care (including large group discussion on stigmatization in care, misinformation about mental illness and confidentiality); overview of the guide and accompanying Master Chart *16 h–17 h*: Small work groups: Role plays on building trust and proper communication with patients
Week 2 (14 h–17 h30)	*Depression*	*To learn*: how to detect signs and symptoms related to depression, as well as current psychosocial stressors; about pharmacological and non-pharmacological interventions for depression; about managing people presenting with signs and symptoms of depression (e.g. to establish a proper follow-up, to engage with family members if appropriate and when/where to refer)	*14 h–15 h*: General lecture using *mhGAP-IG* PowerPoint: overview of depression (including large group discussion on local names and causes); evaluating signs and symptoms of depression, and working with this population (including accompanying video and discussion) *15 h–15 h50*: Small work groups: Role play on evaluation of signs and symptoms of depression, and diagnosing using the guide *15 h50–16 h50*: Treatment, management and follow-up (including large group discussion on myths about types of treatment for depression) *16 h50–17 h30*: Small work groups: Role play on pharmacological and non-pharmacological treatments
Week 3 (14 h–17 h30)	*Psychosis*	*To learn*: how to detect signs and symptoms related to psychosis, and schizophrenia; about pharmacological and non-pharmacological interventions for psychosis, and schizophrenia; about managing people presenting with signs and symptoms of psychosis or schizophrenia (e.g. to establish a proper follow-up, to engage with family members if appropriate and when/where to refer)	*14 h–15 h30*: General lecture using *mhGAP-IG* PowerPoint: overview of psychosis (and schizophrenia) (including large group discussion on causes and current perceptions of these disorders); evaluating signs and symptoms of psychosis/schizophrenia and working with this population (including accompanying video and discussion) *15 h30–16 h*: Small work groups: Role play on evaluation of signs and symptoms of psychosis/schizophrenia, and diagnosing using the guide *16 h–17 h*: Treatment, management and follow-up (including large group discussion on pharmacological/non-pharmacological treatment) *16 h50–17 h30*: Small work groups: Role play on follow-up with patients with psychosis/schizophrenia (including addressing secondary effects of pharmacological treatment)
Week 4 (14 h–17 h30)	*Suicide/self-harm*	*To learn*: how to evaluate thoughts, plans and acts of self-harm by asking appropriate questions; about specific interventions for suicide/self-harm; about managing people presenting with signs and symptoms of self-harm/suicide (e.g. to establish a proper follow-up, to engage with family members if appropriate and when/where to refer)	*14 h–16 h30*: General lecture using *mhGAP-IG* PowerPoint: overview of suicide/self-harm (including large group discussion on myths and importance of addressing suicide/self-harm in practice); evaluating thoughts of self-harm and working with this population (including accompanying video and discussion) *16 h30–17 h*: Small work groups: Role play on evaluation of thoughts self-harm/suicide, and diagnosing using the guide *17 h–17 h30*: Treatment, management and follow-up
Week 5 (14h–17 h)	*Drug/alcohol use disorders*	*To learn*: how to detect signs and symptoms related to substance use disorders; about pharmacological and non-pharmacological interventions for substance use disorders; about managing people presenting with signs and symptoms of substance use disorders (e.g. how to establish a proper follow-up, to engage with family members if appropriate, recognize use patterns and when/where to refer)	*14 h–15 h15*: General lecture using *mhGAP-IG* PowerPoint: overview of alcohol/drug use disorders (including large group discussion on causes and local substances); evaluating signs and symptoms of alcohol/drug use disorders and working with this population *15 h15–16 h*: Small work groups: Role play on evaluation of signs and symptoms of alcohol/drug use disorders, and diagnosing using the guide *16 h–16 h50*: Treatment, management and follow-up *16 h50–17 h30*: Small work groups: Role play on management using non-pharmacological treatment (i.e. brief psychoeducation intervention)
Week 6 (14 h–16 h)	*Support session*	To gain insight/direction on specific mental health cases seen in clinical practice; To learn from colleagues about challenges to appropriate mental health care in clinical practice; To further role plays	*14 h–15 h*: Small work groups: Presentation of mental health cases *15 h–16 h*: Small work groups: Role plays on evaluation of signs and symptoms of all disorders covered during the training, and diagnosing using the guide

### Required adaptation 3: adapting content to context

#### Context's influence on conditions’ specificities

Important observations were made regarding context's influence on conditions’ specificities, thus encouraging changes to standard training material, such as PowerPoints. Discussions and modifications were needed in three principal areas of the standard training: (1) self-harm/suicide; (2) substance use disorders; and (3) the development and use of psychotherapeutic skills, as suggested by certain standard training modules. Changes to standard PowerPoints were made by JS. Adapted PowerPoints were then sent to members of the Ministry of Health and trainer-psychiatrists for final review before training implementation.

The standard training specifies that the most common means of suicide in low- and middle-income countries are the use of firearms and ingestion of pesticides (World Health Organization, 2010). However, in Tunisia, the rate of suicide by firearm is 0.27% given that privately owned guns are rare (Ministère de la santé, 2016). For example, Tunisia ranked 173rd out of an examined 178 countries regarding the number of privately owned guns, and 178th based on the rate of owning a gun (Karp, 2007). In addition, the rate of suicide associated with the ingestion of pesticides in Tunisia is relatively low, at 2.74% (Ministère de la santé, 2016). Changes to the training material thus required the addition of the two most prominent means of completed suicide in Tunisia: hanging (58.63%) and immolation (15.89%) (Ben Khelil *et al.* 2016a; Ministère de la santé, 2016). Hanging is widespread given the accessible and affordability of the means, and immolation has been used especially after the public immolation of Mohamed Bouazizi, sparking the Revolution (Ben Khelil *et al.* 2016a,b; Ben Khelil *et al.* 2017). However, it is important to note that while the rate of completed suicide by ingestion of pesticides is quite low in comparison with hanging and immolation, it was not removed from the training material because it is a prominent means of attempted suicide. Readily available and easily purchased (i.e. often costing <1 Tunisian dinar) pesticides cause concern given rising consultations at emergencies and suicidal tendencies in the country.

In Tunisia, the rise of substance use disorders is worrisome, especially given that these disorders are heavily stigmatized (Unité de Promotion de la Santé Mentale, 2013). Stigmatization encourages healthcare professionals to often dismiss substance use disorders as moral faults. Therefore, it was imperative to add the following information to the standard PowerPoints related to substance use disorders: (1) biological facts about the impact of alcohol and drugs on the brain and how they may cause dependency, especially among those living with certain preconditions; and (2) specific details on substance use disorders in Tunisia. More specifically, given no national epidemiological study on the prevalence of substance use disorders in the country, estimated statistics provided by the Ministry of Health were added to the standard PowerPoints. Such statistics show that of the estimated 350000 people living with substance use disorders in the country, 70% of them are under the age of 35 (Unité de Promotion de la Santé Mentale, 2013). In addition, current drugs in circulation and their local names were shared. These include: opioids (local names: *Buprenorphine* and *Subutex*), cannabis (local name: *Zatla*) and ecstasy (local name: *Fliss*).

Many standard modules of the *mhGAP-IG* selected for training include therapeutic interventions (i.e. behavioural activation, interpersonal therapy, cognitive–behavioural therapy, contingency management therapy, family counselling/therapy, interpersonal psychotherapy or motivational enhancement therapy) as part of the management skills to be developed by trainees. It is important to note, however, that limited trainings on such therapies have only recently been introduced in Tunisia, consequently reserving many of these types of therapeutic interventions to psychosocial care providers, such as psychologists or psychiatrists. Thus, psychotherapy is very rarely conducted by GPs. These therapies were removed from the standard training content, but were mentioned orally to highlight other types of treatment than pharmacological.

GPs in the Greater Tunis area do, however, engage in psychoeducation with people consulting for mental health problems, substance use disorders and suicidal ideation. Thus, during training, appropriate information to be shared with people consulting with mental illness or suicidal ideation, as listed in the standard guide, would be taught and reinforced.

#### Context's impact on the availability of medication

Context plays a significant role on the availability of psychotropic medications in healthcare clinics in the Greater Tunis area. First, while many psychotropic medications listed in the *mhGAP-IG* and the *World Health Organization Model List of Essential Medicines* are available in primary care settings, differing internal procedures on the inclusion of medication in clinics cause uneven distribution and difficulty in prescribing. For example, certain non-standardized procedures were established to counter the stealing of *Trihexyphenidyl* mainly in areas where crime rates were high post-Revolution. In addition, *Benzodiazepines*, despite their availability in certain primary healthcare settings, are very rarely used by GPs. Conditional to their use are the following: a suggested minimal level of mental health training and knowledge of the drugs (which very few GPs attain given limited medical education on pharmacology in Tunisia), or prescription renewal by these trained GPs. Thus, unavailability of needed treatment in primary healthcare settings and unattainable conditions for GPs to be able to prescribe force often unnecessary referrals to specialized or private settings. Information on uneven distribution of medication across healthcare clinics and barriers to prescription if medication is available was included in the training as to highlight health inequity in practice.

Second, stigmatization of substance use disorders has greatly limited the availability of medication for these disorders in primary healthcare clinics, their prescription mainly reserved for emergency settings (Unité de Promotion de la Santé Mentale, 2013). For example, *Naltrexone* is a medication listed in the *mhGAP-IG* for treatment of alcohol dependence. While it is available in Tunisia, it only exists in injectable form, and is mainly utilized by resuscitators in emergency settings. *Acamprosate* and *Disulfiram*, also listed as medications in the *mhGAP-IG* to treat alcohol use disorders, are currently not available in Tunisia. In addition, *Methadone*, used to reduce withdrawal symptoms caused by heroin, is unobtainable in Tunisia. Bringing these deficits to light would be an attempt to show GPs that many cases of substance use disorders may be treated in primary care, given treatment availability and proper support.

#### Context's impact on the availability of community-based mental health services

Community-based mental health resources, ones that promote recovery and reintegration into economic and social activities through supported employment, housing and education opportunities, are important components of the *mhGAP-IG (version 1.0)* (World Health Organization, 2010). However, in Tunisia, while the Ministry of Health aims to support the transition from institutional- to community-based care, most of the mental health budget continues to be used to sustain hospital settings treating mental illness, to the detriment of developing and sustaining community-based mental health resources (World Health Organization and Ministry of Health Tunisia, 2008; Unité de Promotion de la Santé Mentale, 2013). More specifically, little investment in subsidized housing makes affordable housing scarce and difficult to obtain, while supported housing, assisted living facilities and supported employment initiatives are currently not available in the public sector. Only very limited sheltered homes (i.e. a maximum of approximately 200 beds for the entire country, and long ago filled) are available in the public sector for people living with mental illness but without any family support (World Health Organization and Ministry of Health Tunisia, 2008).

In addition, there is a deficit of psychosocial care providers in the country, whose mandate is to help people living with mental illness further develop skills and connect with needed resources in the community. In Tunisia, there are approximately 2.9 psychosocial care providers for 100000 people (Bruckner *et al.* 2011), and they mainly work in institutional/specialized settings or the private sector (World Health Organization and Ministry of Health Tunisia, 2008). To meet current need in Tunisia, however, an estimated minimum of 9.8 psychosocial care providers per 100000 people are encouraged (Bruckner *et al.* 2011), specifically working within the community.

Tunisia's mental health programme at the level of the Ministry of Health was created to point out these deficits in needed community-based mental health resources. The importance of missing community-based mental health resources was therefore highlighted in the adapted training, with the hope of encouraging GPs to also advocate for such services in the Greater Tunis area.

Of note, stigmatization of drug and alcohol use disorders in Tunisia has prevented the development and implementation of a standardized structure of care, beginning in the community, for people living with these conditions (Unité de Promotion de la Santé Mentale, 2013). For example, the *mhGAP-IG* suggests referrals to residential rehabilitation programmes. However, people needing such services in Tunisia are inevitably referred to psychiatric units, emergency medical centres or the private sector. These services are very rarely specialized in the treatment of substance use disorders, as they merely engage in general psychiatric treatments, preventing care from being adequately adapted to those consulting for needed services. In addition, the *mhGAP-IG* suggests referrals to formal support/self-help groups for people living with substance use disorders, useful for peer contact, sharing, support and networking. However, formally, support groups for this population do not exist and are not recognized in Tunisia.

Given the emphasis put on ‘emergency’ care for substance use disorders, and thus short-term follow-up, trainer-psychiatrists sought to help trainees better understand the benefits of developing longer term treatment plans for people presenting with these disorders in primary care, with the support of specialists. The training thus included teachings on scheduling future appointments and building therapeutic alliances. In addition, trainer-psychiatrists insisted that adapted training material include referrals to formal support/self-help groups even though they do not formally exist in the Greater Tunis area, with the hope that this information would encourage GPs to recognize their importance and advocate for their creation.

#### Required adaptations: summary

Required adaptations to the training content and standard programme, as well as the realities that fuelled them, are summarized in Table 2.

**Table 2:** Adaptations made to the standard Mental Health Gap Action Programme (mhGAP) Intervention Guide (IG) to meet realities of the Greater Tunis area (Tunisia)

Required adaptation	Local realities	Implications	Suggested adaptation
*Selecting training modules*	*Context's influence on choice of modules.* The need to address:		
	the rise in anxiety, depressive and substance use disorders, as well as suicide since the 2010-2011 Revolution. the association between schizophrenia and suicide/suicide attempts and reported increase in annual mortality rates associated with schizophrenia.	All needed modules were available, except the training material on conditions specifically related to stress.	General practitioners in charge of continuing medical education in the Greater Tunis area were assigned the role of “tutors” and given access to trainer-psychiatrists for support in filling this knowledge gap during and post-training. Anxiety disorders were covered indirectly in the depression module.
	field observations highlighting that general practitioners may share offices to provide care, were interrupted during consultations by waiting patients, and/or answered phone calls during consultations.	Discussions with trainees on confidentiality and good clinical practices for effective communication and interactions of healthcare professionals with people seeking mental health care were encouraged.	These local realities observed through field observation reinforced the need for the *“General Principles of Care”* module.
*Developing a training format*	*Context's influence on training model and schedule.* General practitioners have a restrained work schedule. Deficits in continuing mental health training programs in the Greater Tunis area. Psychiatrists in Tunisia have time constraints and a heavy workload. Letter written by general practitioners to refer patients to more specialized care. Consultations with patients in Tunisia are conducted in Tunisian Arabic.	General practitioners conduct clinical practice from 8 h-14 h, Monday to Saturday. These deficits create a high demand for training. Ongoing supervision post-training, as suggested by the standard programme, was not feasible. Challenges for trainees given short training programme, and often long referral procedure by letter. Role plays would thus be more realistic if conducted in Tunisian Arabic.	The training was designed to include only 1 session per week. The general lecture was conducted with all trainees, but small groups for role plays and more in-depth discussion were created. A 2-hour support session post-training was offered. In addition, the role of tutors was extended: they would provide guidance to trainees during and after training; if needed, they had access to trainer-psychiatrists during and after training for more in-depth questioning; and they would be able to organize support sessions with trainees post-training given their active role in continuing medical education. Trainer-psychiatrists provided their numbers to trainees, to facilitate referrals (if needed) during training. Tunisian trainers translated role plays into Tunisian Arabic, and simulation of consultations were conducted in this language by general practitioners during role plays.
*Altering content based on context.*	*Context's impact on conditions’ specificities.* *Suicide* Means of suicide are affected by availability and affordability of the means, and political context. *Substance use disorders* Substance use disorders are heavily stigmatized in the country. Rise of substance use and substance use disorders in Tunisia. *Psychotherapies* Psychotherapies are usually considered the responsibility of psychosocial care providers, not general practitioners.	Main means of suicide in Tunisia are hanging and immolation, not by use of firearms or ingestion of pesticides (ingestion of pesticides is a common way of attempted suicide). General practitioners do not always acknowledge substance use disorders as an ‘illness.’ No national prevalence of substance use disorders in Tunisia is available, only estimated statistics. Rise of substance use disorders caused by specific substances, which have local names. General practitioners usually engage in active listening and psychoeducation.	Training included local means of suicide/suicide attempts, but also highlighted the possibility of completed suicide by ingestion of pesticides given their availability and affordability. Information on the effect of drugs and alcohol on the brain and what may cause dependency was added to the training. Estimated statistics by the Ministry of Health were included in the training on substance use disorders to familiarize trainees with the realities associated with these disorders in Tunisia. General practitioners were informed of the local names of substances. Suggested therapies were removed from the standard training content but were mentioned orally to highlight other types of treatment than pharmacological. General practitioners do engage in psychoeducation. Therefore, appropriate information to be shared with people consulting for mental illness or suicidal ideation, as listed in the standard guide, was taught and reinforced.
	*Context's impact on availability of medication.* Listed medication in the standard training and *World Health Organization Model List of Essential Medicines* are available in Tunisia, but there are different internal procedures for the availability and prescription of these medications within healthcare clinics. Substance use disorders are heavily stigmatized in Tunisia. *Context's impact on availability of community-based mental health services.* While there is a budget for mental health prevention activities, most mental health funding is allocated to sustain institutionally-based resources. Substance use disorders are heavily stigmatized in Tunisia.	There is an uneven distribution of needed medication across healthcare clinics and the ability to prescribe it is sometimes challenging. Medications to treat these disorders, if available, are mainly available in emergency settings, hospital settings, or the private sector. There are deficits in community-based services that promote recovery and reintegration. For people living with substance use disorders, there are no standardized structures of care rooted in the community or formal support/self-help groups available. This encourages greater short-term follow-up.	To highlight health inequity, the uneven distribution of essential medicines and the conditions to prescribe them for people living with mental health problems in primary care settings was included in the training. The monopoly of these medicines in emergency, hospital, or private settings were highlighted, but general practitioners’ role in treatment, if resources and support were available, was emphasized. Missing community-based services were included in the training to highlight their importance and encourage general practitioners to advocate for them. Training included ways in which general practitioners can manage this population over the longer term, and the need for formal support/self-help groups and residential rehabilitation services.

## Discussion

To our knowledge, this is the first attempt to adapt a training based on the *mhGAP-IG* in Tunisia, and one of the first in a French-speaking nation (Keynejad *et al*. 2017; Spagnolo *et al.* 2017). The decision to implement and adapt a mental health training programme in Tunisia was in direct response to the current health system reform seeking to further develop proximity health services (World Health Organization and Ministry of Health Tunisia, 2008; Unité de Promotion de la Santé Mentale, 2013; Comité technique du dialogue societal, 2014), and facilitate the integration of mental health into primary care, an international effort (World Health Organization, 2010; Cohen *et al.* 2014; Prince *et al.* 2014).

The training's adaptation to the Greater Tunis area, which involved multiple stakeholders and processes, such as validation of materials, discussions and field observations, is one example of the ways in which the standard *mhGAP-IG* training material and programme can be adapted to meet local needs. The process highlighted that context has a direct impact on modules selected for training, ways in which the programme is to be designed and offered, conditions’ specificities, availability of psychotropic medications in healthcare clinics, and availability of community-based mental health services that aim to promote recovery and reintegration. Without the involvement of local decision-makers, psychiatrists and GPs, the production of location-specific training material and the creation of a realistic programme that can be sustained or reproduced would not have been possible.

Uncovering systemic gaps in primary mental health care was, in our opinion, one of the most important outcomes of the adaptation process. These include lack and uneven distribution of psychotropic medications across healthcare clinics in the Greater Tunis area, as well as deficits in community-based mental health services for people living with mental illness. The adaptation process was tailored by members of the Ministry of Health in part to make clearer where there are gaps in service delivery.

All stakeholders aimed to address systemic barriers to effective mental health care in the adapted training programme for the Greater Tunis area by: (1) emphasizing primary care as a plausible setting in which mental illness may be detected, treated and managed; (2) developing a practical and feasible structure to support trainees during and post-training; and (3) highlighting the needed but unavailable public resources explicitly listed in the standard *mhGAP-IG.* We hoped that highlighting unavailable resources would help improve trainees’ attitudes towards mental illness and mental health integration within primary care, empower trainees to advocate for the uniform availability of psychotropic medications, and encourage trainees to campaign for the funding, development and implementation of non-existent community-based mental health services in the public sector.

We acknowledge that encouraging GPs to advocate for mental health services within primary or community-based settings all the while building their mental health capacity with an adapted training is not enough to foster the programme's success and sustainability in the Greater Tunis area. First, adapting a training programme before implementation becomes redundant if decision-makers outside of the realm of mental health do not acknowledge the importance of funding non-specialized mental health resources (Jacob, 2011). In other words, ‘policy makers need to be convinced about the reality of unmet needs and the fact that simple and affordable interventions are available’ (Gureje *et al.* 2015). In Tunisia, the Committee for Mental Health Promotion was created to ensure that mental health is a priority in Ministry. The development of the *National Strategy for the Promotion of Mental Health in Tunisia*, a response of this Committee, also confirms that mental health is being recognized in policy (Unité de Promotion de la Santé Mentale, 2013). However, while political recognition is important, it is essential to ensure that adequate funding continues to be invested as to facilitate the transition from institution to community-based care in the country. More specifically, appropriate funding, reflecting the country's burden caused explicitly by mental disorders, substance use disorders and suicide, should be invested as to develop and sustain the needed but unavailable public resources, examples of which are listed in the standard *mhGAP-IG.* Without adequate and continued funding allocated to non-specialized mental health resources within the community, this adapted training, and future ones under the auspices of the Ministry of Health, will most likely be unsuccessful and unsustainable.

Secondly, adapted mental health training programmes may become unsuccessful if people living with mental health problems or substance use disorders do not access developed services, resources or GPs who have been trained in effective mental health care. Therefore, anti-stigma interventions targeting the public have been declared a priority in global mental health (Wainberg *et al.* 2017). In Tunisia, this role has been traditionally left to individual, non-governmental organizations, without clear implementation guidelines or follow-up (Unité de Promotion de la Santé Mentale, 2013). However, the recent publication of the *National Strategy for the Promotion of Mental Health in Tunisia* includes anti-stigma initiatives under the mandate of the Committee for Mental Health Promotion, thus ensuring more standardized implementation and follow-up (Unité de Promotion de la Santé Mentale, 2013).

In recent years, the Committee for Mental Health Promotion has attempted to target the echoes of stigma attached to mental illness by actively speaking about mental health through mass media. More specifically, members of the Committee regularly organize interviews with popular Tunisian channels and national television chains to discuss important topics, such as depression and suicide. In addition, in 2017, *World Mental Health Day* was celebrated, in collaboration with the World Health Organization office in Tunisia and members of the Ministry of Health, by encouraging directors of governorates to organize events on depression for primary healthcare professionals across the country.

Other initiatives to decrease mental health stigma and encourage prevention include the development of national suicide prevention and substance use strategies. The development of these important documents is a collaboration between multiple stakeholder groups, to reflect the intersectionality of these issues. In addition, equipped with lessons learned from this adaptation and implementation, trainings based on the *mhGAP-IG* in other areas of Tunisia are envisioned.

### 

#### Limitations

Limitations of the training programme are worthy of note. First, due to financial and human constraints, it was not possible to create a new guide for trainees, comprising the adaptations made to standard content. To compensate, adaptations were made to material used in training sessions, such as PowerPoints. A second limitation to the training is the little emphasis placed on psychotherapies, given that these are considered the responsibility of psychosocial care providers in Tunisia. Lastly, in our opinion, it would have been beneficial to involve, during discussion about mental health needs and gaps, personnel beyond psychiatrists and GPs. Diverse types of personnel could help highlight the mental health realities in the Greater Tunis area from complimentary lenses.

## Conclusion

The adaptation of a training based on the *mhGAP-IG* to the Greater Tunis area of Tunisia was needed for location-specific use. The adaptation process highlighted required changes to the standard training and programme, influenced by contextual realities. However, it is important to note that systemic issues, such as the lack and uneven distribution of medication, echoes of stigmatization towards mental illness and the field of mental health, and the unavailability of community-based mental health services that promote recovery and reintegration, may hinder the successful implementation and sustainability of the adapted programme. These barriers are important to consider as they may perpetuate the growing mental health treatment gap. Therefore, systemic barriers must inevitably be addressed by initiatives beyond the adapted training programme.
